# 人血清中30种磷脂酰胆碱与溶血磷脂酰胆碱同系物的液相色谱-串联质谱测定及其与冠心病的关联分析

**DOI:** 10.3724/SP.J.1123.2025.08011

**Published:** 2026-03-08

**Authors:** Wenyu LI, Zhaoyang LIU, Jun DONG, Ruiyue YANG, Hongxia LI, Wenxiang CHEN, Siming WANG

**Affiliations:** 北京医院实验研究部 国家老年医学中心，国家卫生健康委老年医学重点实验室，中国医学科学院老年医学研究院，北京 100730; Department of Experimental Research，Beijing Hospital，National Center of Gerontology，Key Laboratory of Geriatrics of National Health Commission，Institute of Geriatric Medicine，Chinese Academy of Medical Sciences，Beijing 100730，China

**Keywords:** 磷脂酰胆碱, 溶血磷脂酰胆碱, 动脉粥样硬化, 冠心病, 液相色谱-串联质谱, 脂质组学, phosphatidylcholine, lysophosphatidylcholine, atherosclerosis, coronary artery disease, liquid chromatography-tandem mass spectrometry （LC-MS/MS）, lipidomics

## Abstract

磷脂酰胆碱和溶血磷脂酰胆碱同系物与冠状动脉粥样硬化密切相关，准确测定其含量可以为临床上冠心病的诊断和预后提供重要依据。本研究建立了一种基于液相色谱-串联质谱的分析方法，仅需10 μL人血清即可同时实现30种磷脂酰胆碱和溶血磷脂酰胆碱同系物的准确测定。采用甲醇-乙腈-甲基叔丁基醚-水作为萃取体系，选用XBridge C18色谱柱，以乙腈-水（1∶1，体积比）混合溶液和异丙醇溶液作为流动相，两相均含有7.5 mmol/L甲酸铵与0.15%（体积分数）甲酸，进行梯度洗脱分离，采用电喷雾离子源，正离子多反应离子检测模式。经验证，该方法线性关系良好，平均线性相关系数≥0.999 7，线性范围为0.125~100 μg/mL，检出限和定量限分别为0.01~1.94 μg/mL和0.03~6.48 μg/mL，回收率为85.4%~114.3%，日内精密度和日间精密度分别不大于4.6%和12.6%。使用本方法测定了临床上110名接受冠状动脉造影术志愿者的血清样本，磷脂酰胆碱同系物平均人群质量浓度为526.80 μg/mL，溶血磷脂酰胆碱同系物平均人群质量浓度为73.67 μg/mL。Spearman相关性检验分析发现，磷脂酰胆碱和溶血磷脂酰胆碱同系物与冠心病严重程度以及相关临床生化、血脂代谢指标存在密切关联，提示其可作为临床冠心病的潜在相关代谢物。本研究针对临床分析需求设计，具有血清用量少、操作简便、响应优良等特点，可高效实现人血清中30种磷脂酰胆碱和溶血磷脂酰胆碱同系物的测定，为探究二者与冠心病的关联及生物标志物转化应用提供了重要参考。

## Abstract (en)

Phosphatidylcholine （PC） and lysophosphatidylcholine （LPC） homologues are closely associated with coronary atherosclerosis. Accurate determination of their contents can provide an important basis for the clinical diagnosis and prognosis of coronary artery disease （CAD）. In this study， an analytical method based on liquid chromatography-tandem mass spectrometry was established， which enabled the simultaneous and accurate determination of 30 PC and LPC homologues using only 10 μL of human serum. Methanol-acetonitrile-methyl *tert*-butyl methyl ether-water was used as the extraction system， and an XBridge C18 column was selected as the stationary phase. The mobile phase consisted of an acetonitrile-water mixture （1∶1， volume ratio） and isopropanol， both containing 7.5 mmol/L ammonium formate and 0.15% （volume ratio） formic acid， and gradient elution was adopted for separation. Detection was performed using an electrospray ionization source in the positive ion mode with multiple reaction monitoring. Method validation results showed that the method exhibited a good linear relationship， with an average linear correlation coefficient of ≥0.999 7 over a linear range of 0.125-100 μg/mL. The limits of detection and limits of quantification were 0.01-1.94 μg/mL and 0.03-6.48 μg/mL， respectively. The recoveries ranged from 85.4% to 114.3%， while the intra-day precision and inter-day precision were no more than 4.6% and 12.6%， respectively. Serum samples from 110 clinical volunteers who underwent coronary angiography were determined using this method. The average population concentration of PC homologues was 526.80 μg/mL， and that of LPC homologues was 73.67 μg/mL. Spearman correlation analysis revealed that PC and LPC homologues were closely correlated with the severity of CAD， as well as with related clinical biochemical and lipid metabolism indicators， suggesting that they could serve as potential CAD-related metabolites in clinical practice. Designed to meet clinical analysis needs， this method features small serum sample volume， simple operation， and excellent response. It can efficiently determine 30 PC and LPC homologues in human serum， providing an important reference for exploring the association between these two lipid classes and CAD， as well as the translational application of related biomarkers.

冠状动脉粥样硬化性心脏病（冠心病， coronary artery disease， CAD）是全世界范围内危害人类健康最严重的疾病之一，并对家庭、社会及公共医疗体系构成了沉重负担^［1］^。CAD逆转困难，及早发现和鉴定其主要危险因素并进行有效干预是降低CAD发病率和死亡率的重要手段^［2］^。目前临床上用于评价CAD风险的传统危险因素主要是血清总胆固醇（TC）、甘油三酯（TG）、脂蛋白胆固醇等血脂水平。虽然传统血脂指标在CAD临床防治方面发挥了重要作用，但临床上仍然有相当一部分CAD患者未表现出明显的血脂异常^［3］^。部分急性心梗病人血脂处于正常水平，甚至有人在降脂治疗期间突发急性心梗，提示传统危险因素仍存在较大的残余风险^［4］^。因此，寻找新的CAD危险因素生物代谢物、提升其风险预示能力，对CAD防治监测具有重要意义。

多项临床及流行病学研究均提示^［5］^，CAD的发生发展与脂质代谢紊乱密切相关。磷脂酰胆碱（phosphatidylcholines， PC）与其水解产物溶血磷脂酰胆碱（lysophosphatidylcholine， LPC）是脂代谢中的一类关键分子。一项队列研究表明，血清PC水平降低（尤其是多不饱和亚型）与动脉粥样硬化相关心血管事件风险升高相关，且该关联独立于传统血脂指标^［6］^；此外另一项报告表明，与对照组相比，一些血清LPC与有症状的动脉粥样硬化患者的部分指标呈负相关^［7］^。与之呼应，在一项长达18年的前瞻性随访研究中，基于神经酰胺及PC、LPC等脂质分子构建的心血管风险评分，被证实可有效预测心血管疾病事件的发生风险^［8］^。该研究进一步揭示，PC 16：0/16：0水平与CAD风险呈正相关，且其与心血管疾病及脑卒中死亡风险的关联强度更为显著。Paavola等^［9］^亦发现，在CAD患者及代谢综合征人群中，PC 16：0/16：1、16：0/18：1水平分别较对照组均有所升高。与之形成鲜明对比的是，LPC 18：2水平不仅未随冠心病进展升高，反而呈现与疾病风险显著负相关的趋势，直接体现了不同PC及LPC同系物在心血管调节中可能存在功能方向性差异。这一亚型特异性差异在Fernandez等^［10］^的研究中得到进一步印证，他们发现LPC 16：0和LPC 20：4与心血管疾病风险降低显著相关，提示特定LPC同系物可能具有心血管保护作用。上述结果表明，不同亚型的PC和LPC在CAD的诊断与预后评估中扮演着异质性角色，其与CAD的关系仍需深入探究。

近年来，液相色谱-串联质谱分析技术已应用于脂质组学研究，为PC和LPC同系物的鉴定与定量提供了技术支撑，但其方法多以合并测定多类脂质为导向，侧重整体脂质谱的解析^［11］^。这一特点导致临床研究中PC、LPC的纳入范围常显局限。近期一项针对糖尿病人群的脂质分析方法虽涵盖310种小分子脂类，但其针对PC、LPC仅选取PC 36：1、LPC 18：2等少数亚型进行关联分析^［12］^，远未覆盖该家族中可能参与CAD病理过程的更多潜在亚型。鉴于不同种类的PC、LPC可能在CAD发生发展过程中扮演着不同的角色，因此建立覆盖面广、能够适用于临床人群队列分析需求的PC、LPC分析方法便显得尤为重要。此外，常规脂质组学方法的样本消耗量多处于50~200 μL范围^［13］^，并不特别满足临床人群队列研究对于微量样本的需求。针对上述瓶颈，本研究建立了一种精准、简便的分析方法，仅需微小样本量即可实现多种PC、LPC同系物的覆盖。我们用所建方法在冠脉造影人群队列中研究了PC、LPC与CAD危险因素的关系，为CAD风险分层及磷脂标志物的临床转化提供参考依据。

## 1 实验部分

### 1.1 仪器、试剂与材料

LC-MS/MS系统由Agilent 1260高效液相色谱仪（美国Agilent 公司）、SCIEX QTRAP 5500质谱仪和Analyst 1.6.3数据处理软件（美国SCIEX公司）组成。血液混匀器（无锡杰瑞安仪器设备有限公司），Sorvall ST 8R高速冷冻离心机和真空烤箱（德国Thermo Scientific公司），振荡器（美国Leap Scientific公司），MicroLab 500型稀释器（美国Hamilton公司），BT124S分析天平（德国Sartorius公司）。

标准品（纯度>99%）：LPC 14：0、LPC 15：0、LPC 16：0、LPC 18：0、LPC 20：0、LPC 24：0、LPC 18：1、PC 7：0/7：0、PC 9：0/9：0、PC 14：0/14：0、PC 14：0/18：0、PC 14：1/14：1、PC 15：0/15：0、PC 16：0/16：0、PC 16：0/18：0、PC 16：0/18：1、PC 16：0/18：2、PC 16：1/16：1、PC 17：0/17：0、PC 18：0/18：0、PC 18：0/18：1、PC 18：0/18：2、PC 18：1/14：0、PC 18：1/18：1、PC 18：2/18：2、PC 19：0/19：0、PC 20：0/20：0、PC 21：0/21：0、PC 22：0/22：0（美国Sigma-Aldrich 公司）。内标物（纯度>99%）：d31-LPC 16：0、d35-LPC 18：0、d5-LPC 15：0（美国Sigma-Aldrich 公司）、d9-PC 18：0、d4-PC 14：0（英国Cambridge Isotope Laboratories， Inc.）。

质谱纯甲醇、异丙醇、乙腈和甲基叔丁基醚购自美国Thermo Fisher Scientific公司。质谱纯甲酸铵、甲酸购自美国Sigma-Aldrich公司。超纯水由实验室纯水仪（德国Merck Millipore公司）自制。

### 1.2 血清样本

征集110名北京医院心内科行冠状动脉造影的志愿者（年龄50~70岁，男性64名，女性46名），采集静脉血于促凝剂采血管中，在4 ℃下以4 500 r/min离心10 min，取出上层血清分装于冻存管中，置于-80 ℃保存。临床常规生化检验指标部分由临床检验数据系统直接调取，部分由本实验室既往研究测得^［14，15］^。本研究获得北京医院医学伦理委员会批准（2016BJYYEC-121-02），志愿者均签署了知情同意书。

### 1.3 实验方法

#### 1.3.1 标准溶液、内标溶液的配制

精密称取各标准品。由于所有标准品纯度均大于99%，为简化实验操作，配制标准溶液时直接以称量质量计算含量，不再进行纯度折算。以甲醇为溶剂，精密配制质量浓度为0.125、0.25、1.00、5.00、10.00、25.00、50.00、100.00 μg/mL的一系列混合标准溶液，并分装于安瓿瓶中，-80 ℃保存。5种内标物质用甲醇配制成终质量浓度为5 μg/mL的混合溶液，于-80 ℃保存。

#### 1.3.2 样本制备

将血清样本、标准溶液和内标溶液解冻、均质化并平衡至室温。使用稀释器依次精密吸取10 μL标准溶液或血清样本到安瓿瓶中，并加入10 μL内标混合溶液，随后依次加入400 μL甲醇-乙腈混合液（1∶1， 体积比）、1 200 μL甲基叔丁基醚溶液（含体积分数0.1%甲酸），经振荡器振荡10 min，之后加入1 000 μL超纯水，混匀，4 ℃下以4 500 r/min离心10 min，吸取600 μL上清液至进样瓶中，经氮气吹干后，用500 μL初始比例流动相复溶，采用LC-MS/MS系统进行分析。

#### 1.3.3 色谱条件

采用Waters X Bridge C18色谱柱（100 mm×2.1 mm，3.5 μm）。流动相A：乙腈-水（1∶1，体积比）；流动相B：异丙醇；两相均含7.5 mmol/L甲酸铵与0.15%（体积分数）甲酸，以300 μL/min的流速进行梯度洗脱分离，梯度如下：0~4 min，32%B；4~8 min，32%B~84%B；8~17 min，84%B；17~19 min，84%B~32%B；19~25 min，32%B。柱温40 ℃，进样体积2 μL。

#### 1.3.4 质谱条件

质谱扫描模式采用电喷雾离子源（ESI），使用MRM模式。将PC、LPC同系物名称、离子通道监测信息、离子化条件、源气参数条件等优化好的参数输入方法列表，喷雾气、辅助加热气、气帘气和碰撞气均为氮气，分别设置为448.16 kPa、448.16 kPa、158.58 kPa和Medium，喷雾电压5 500 V，离子源温度600 ℃。其他质谱参数见表1。

**表 1:** PC、LPC同系物以及内标物质的质谱参数

Name	RT/min	Precursor ion （*m/z*）	Product ion （*m/z*）	DP/V	EP/V	CE/eV	CXP/V	IS
**PC**								
16∶0/18：0	13.7	762.8	184.2	40	9	38	15	d9-PC 18：0
			86.1	40	9	50	15	
16：0/16：0	13.3	734.5	184.0	83	10	40	17	d9-PC 18：0
			86.1	85	10	53	23	
16：0/18：2	12.9	758.0	183.9	60	10	38	25	d9-PC 18：0
			86.0	60	10	50	25	
18：0/18：0	14.2	790.7	184.1	60	10	40	20	d9-PC 18：0
			86.2	60	10	54	20	
18：1/18：1	13.8	786.6	184.1	50	10	38	20	d9-PC 18：0
			86.0	46	10	50	20	
18：0/18：1	13.7	788.6	184.0	50	10	38	20	d9-PC 18：0
			86.2	50	10	50	15	
14：1/14：1	11.7	674.5	184.0	40	10	35	15	d4-PC 14：0
			86.1	40	10	48	20	
20：0/20：0	15.4	846.7	184.1	40	10	47	20	d9-PC 18：0
			86.2	40	10	53	20	
9：0/9：0	4.4	538.3	184.0	40	10	30	15	d4-PC 14：0
			86.2	40	10	40	15	
7：0/7：0	1.6	482.3	184.1	70	10	29	15	d4-PC 14：0
			86.0	70	10	40	15	
18：1/14：0	11.9	732.6	184.0	50	10	39	20	d4-PC 14：0
			86.1	48	10	50	18	
18：0/18：2	13.3	789.6	184.1	40	10	41	20	d9-PC 18：0
			86.3	40	10	54	16	
18：2/18：2	12.7	782.6	184.1	40	10	39	20	d9-PC 18：0
			86.0	40	10	50	20	
15：0/15：0	12.9	706.5	184.1	80	10	29	15	d4-PC 14：0
			86.2	70	10	42	16	
16：1/16：1	12.5	730.5	184.0	80	10	37	17	d9-PC 18：0
			86.0	50	10	50	20	
16：0/18：1	13.3	760.2	184.1	80	10	36	20	d9-PC 18：0
			86.3	70	10	47	15	
19：0/19：0	14.7	818.7	184.1	30	10	31	17	d9-PC 18：0
			86.1	40	10	41	23	
21：0/21：0	16.1	874.7	184.1	30	10	46	20	d9-PC 18：0
			86.1	30	10	58	20	
22：0/22：0	17.1	902.8	184.2	35	10	47	20	d9-PC 18：0
			86.2	30	10	58	20	
14：0/14：0	12.5	678.6	184.1	60	10	36	20	d4-PC 14：0
			86.2	60	10	50	25	
14：0/18：0	13.2	734.5	184.2	30	10	38	16	d4-PC 14：0
			86.4	40	10	50	20	
17：0/17：0	13.7	762.6	184.2	40	10	40	20	d9-PC 18：0
			86.3	40	10	55	20	
**LPC**								
14：0	3.1	468.3	184.1	80	13	33	14	d5-LPC 15：0
			104.1	80	13	40	13	
15：0	4.6	482.3	184.1	80	10	34	20	d5-LPC 15：0
			104.1	80	10	40	20	
16：0	5.9	496.4	184.0	80	9	35	15	d31-LPC 16：0
			104.0	80	9	40	14	
18：0	10.1	524.4	184.0	70	8	35	15	d35-LPC 18：0
			104.0	70	8	44	14	
20：0	11.3	552.5	184.1	70	7	37	21	d35-LPC 18：0
			104.0	70	7	46	18	
24：0	12.4	608.5	184.1	70	6	40	20	d35-LPC 18：0
			104.0	70	6	50	15	
18：1	6.7	522.4	184.0	70	9	38	15	d35-LPC 18：0
			104.0	70	10	45	15	
18：2	4.1	521.4	184.0	70	8	40	15	d35-LPC 18：0
			104.0	70	10	50	15	
**IS**								
d4-PC 14：0	12.4	682.6	188.0	60	10	36	15	
			86.0	60	10	48	15	
d9-PC 18：0	14.2	799.7	193.0	60	10	40	20	
			86.3	60	10	50	20	
d5-LPC 15：0	4.4	487.4	184.0	80	11	35	24	
			104.0	80	9	40	20	
d31-LPC 16：0	5.8	526.6	184.0	70	6	38	14	
			104.0	70	8	45	15	
d35-LPC 18：0	10.0	558.6	184.2	70	8	37	15	
			104.2	70	8	45	20	

DP： declustering potential； EP： entrance potential； CE： collision energy； CXP： cell exit potential； RT： retention time.

### 1.4 样本测定

用建立的LC-MS/MS法测定110名心内科行冠状动脉造影术的志愿者血清PC、LPC含量，采用IBM SPSS Statistic 26.0（美国IBM公司）软件进行数据统计学分析处理。采用Spearman相关性分析、*t*-检验等方法分析血清PC、LPC与CAD危险因素及相关临床生化指标的关系。定义*P*<0.05为具有统计学显著意义。

## 2 结果与讨论

### 2.1 样品前处理优化

#### 2.1.1 萃取体系

基于PC、LPC同系物的理化性质，本研究系统评估了不同溶剂萃取体系（包括单相纯溶剂体系异丙醇、乙腈、甲醇以及多相萃取体系甲醇-甲基叔丁基醚-水、乙腈-甲基叔丁基醚-水、甲醇-乙腈-甲基叔丁基醚-水）对目标物的提取效率。实验中以总峰面积作为评估指标，其代表了所有目标代谢物特征离子对应的峰面积总和，能综合反映不同实验条件对萃取效果的影响。结果表明，在单相溶剂体系中，异丙醇和甲醇的萃取效率相当，并且显著优于乙腈。进一步对比多相萃取体系发现，当采用甲醇-乙腈体系作为液-液萃取的沉淀剂时，待分析物提取效率最优（图1a）。乙腈作为强沉淀剂，虽能使血清蛋白快速变性沉淀，但单独使用乙腈会导致沉淀过程过快，使PC、LPC类分析物未能与结合蛋白充分解离；而甲醇-乙腈体系通过调控沉淀速率，在有效去除蛋白质的同时，确保了目标分析物的高效释放。另外，甲基叔丁基醚的存在不仅能有效促进相分离，还可推动目标物从水相向有机相转移，减少基质干扰进而提升萃取效率。同时，甲基叔丁基醚沸点低、易挥发，便于后续吹干复溶等样本处理操作。综上，选用甲醇-乙腈混合液-甲基叔丁基醚-水体系。

**图1:**
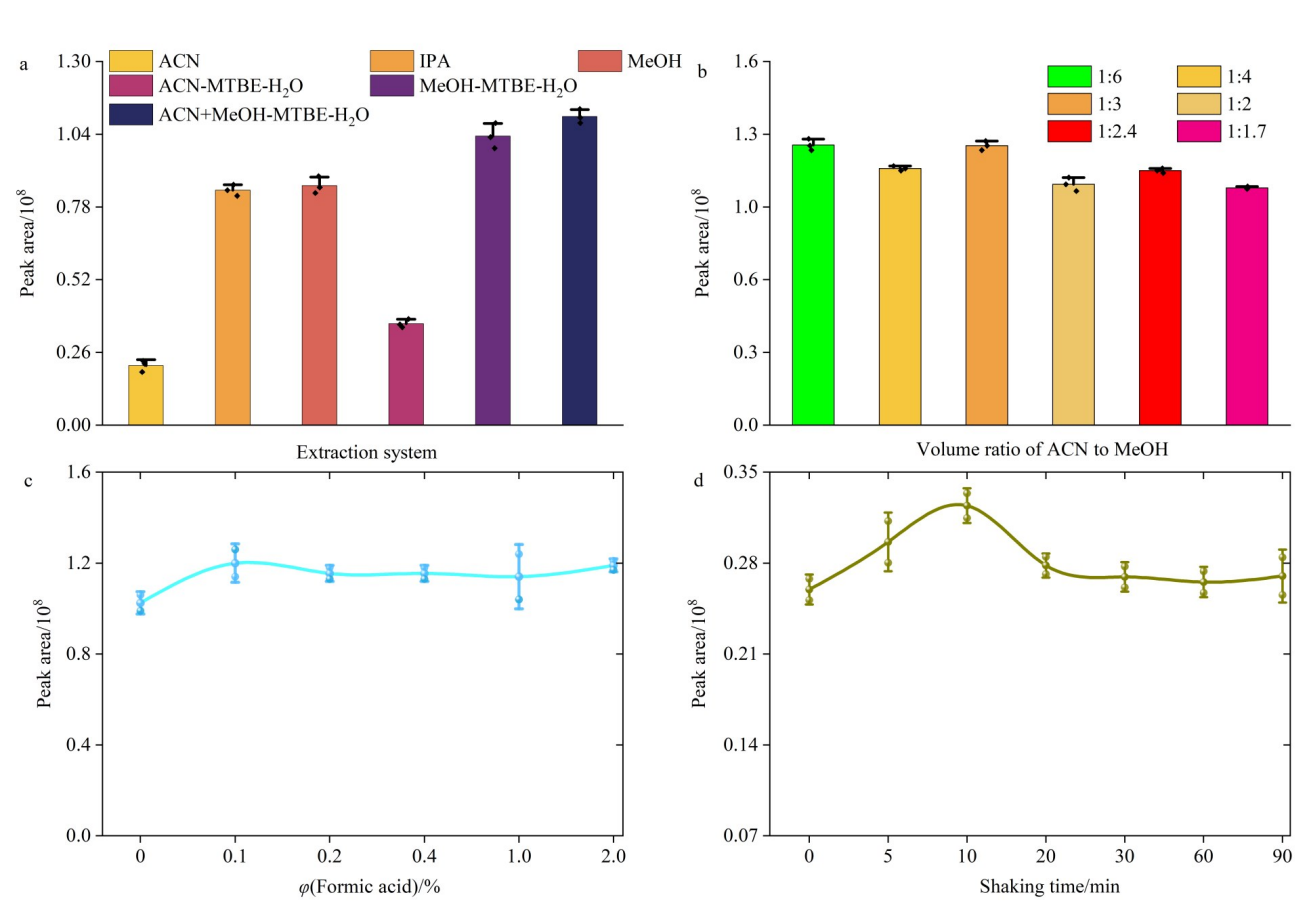
（a）萃取体系、（b）乙腈-甲醇体积比、（c）甲基叔丁基醚中甲酸体积分数、（d）振荡时间对待测物总峰面积的影响（*n*=3）

选定体系后进一步优化沉淀剂和醚的比例，图1b显示当沉淀剂与甲基叔丁基醚的比例为1∶3时，目标物提取效率最佳。

由于弱酸有利于提高脂质的提取和信号响应，我们在甲基叔丁基醚中加入不同体积分数甲酸（0%、0.1%、0.2%、0.4%、1%、2%），观察其对萃取效果的影响。结果显示，当甲酸体积分数为0.1%时目标物提取效果最佳（图1c）。

#### 2.1.2 振荡时间

振荡时间对蛋白质沉淀及脂质提取效率存在显著影响。为评价方法提取效率，本研究于振荡 0、5、10、20、30、60、90 min时取样，比较不同条件下目标物的总离子流峰面积以评价提取效率。结果显示，10 min时效果较优。最终确定的萃取条件如下：10 μL血清依次加入400 μL甲醇-乙腈混合液，1 200 μL甲基叔丁基醚（含0.1%甲酸），1 000 μL水，振荡10 min后离心取上清液用于后续分析。

### 2.2 色谱条件的优化

#### 2.2.1 色谱柱的选择

比较了两种色谱柱：Waters XBridge C8（100 mm×2.1 mm，3.5 μm）和Waters XBridge C18（100 mm×2.1 mm，3.5 μm）。相比于C8色谱柱，C18色谱柱在调整流动相梯度后，目标物保留时间更适宜，同系物间分离度更好，且峰形更优、信号响应更强。尽管C18色谱柱的运行时间略有延长，但结合优化流动相条件后，其时长处于可接受范围。综合考虑色谱峰形、保留时间、分离度、信号响应等因素，选择C18色谱柱。

#### 2.2.2 流动相条件

为保持一定的离子强度，减少拖尾，改善峰形，向流动相中加入一定浓度甲酸和甲酸铵。分别比较不同甲酸/甲酸铵含量对峰形状的影响。结果如图2所示，当甲酸体积分数为0.15%，甲酸铵浓度为7.5 mmol/L时，30种目标物的总离子流峰面积最佳，信号响应也较好。

**图2:**
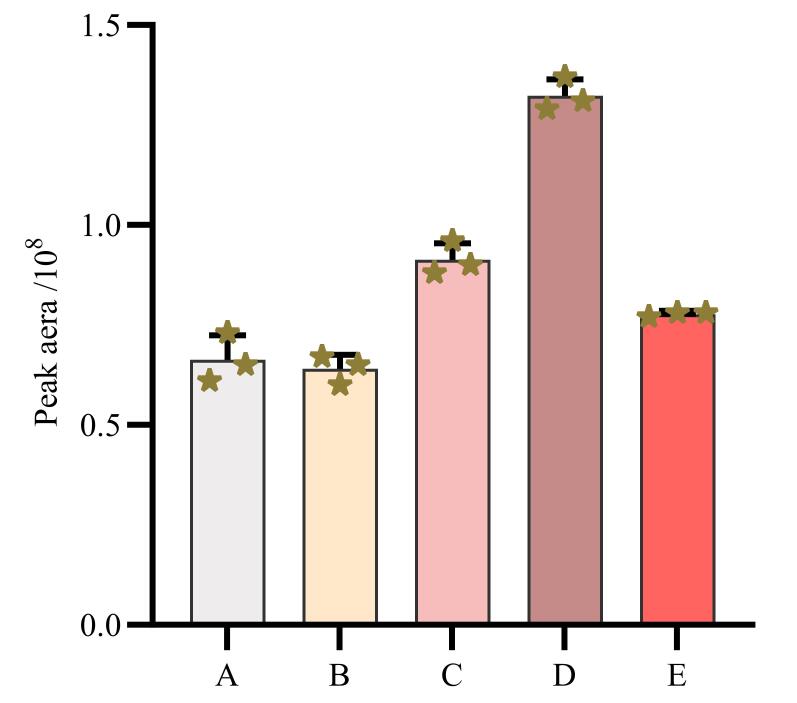
流动相中甲酸体积分数和甲酸铵浓度对目标物总峰面积的影响（*n*=3）

### 2.3 质谱条件优化

在ESI正离子模式下，采集PC、LPC同系物和内标的一级、二级扫描质谱图。根据一、二级质谱信息可推断确定PC、LPC同系物可用于定量监测的母离子为［M+H］^+^离子，碎片离子包括质子化磷酸胆碱头基离子、C_5_H_12_N^+^及C_5_H_14_NO^+^离子等（表1）。每种PC和LPC均至少选取两条离子通道，优先以丰度最大的质子化磷酸胆碱头基特征子离子（*m/z* 184）作为定量离子，选取C_5_H_12_N^+^（*m/z* 86）子离子作为PC定性离子、C_5_H_14_NO^+^（*m/z* 104）子离子作为LPC定性离子。对所选PC和LPC各离子通道的母离子、子离子、去簇电压、入口电压、碰撞能和出口电压等参数进行优化，优化后的质谱条件见表1。图3展示了人血清中丰度较高的PC 16：0/16：0、LPC 16：0为示例的二级质谱扫描图以及PC、LPC同系物的通式及其质谱碎裂规律示意图。

**图3:**
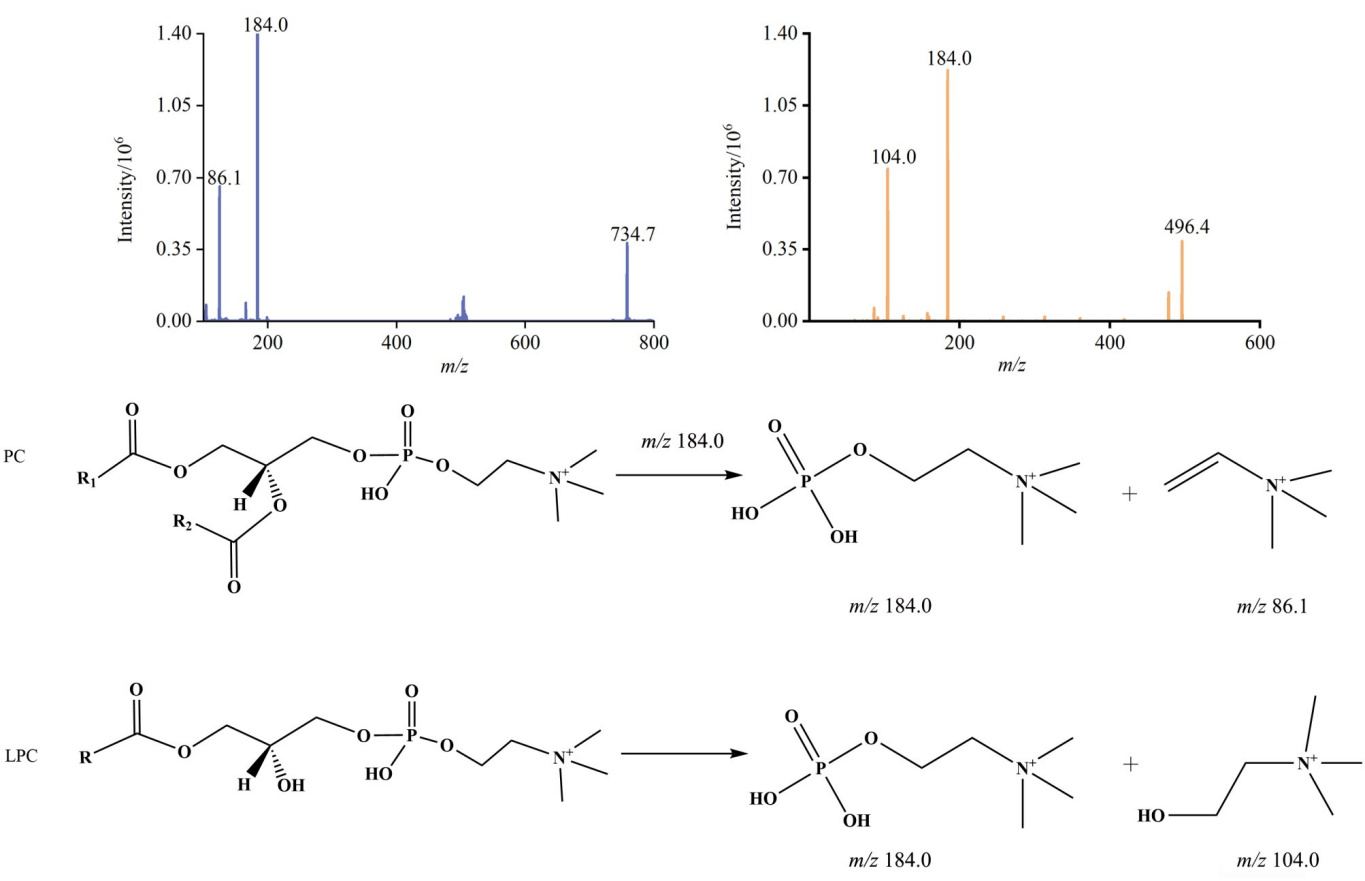
PC 16：0/16：0和LPC 16：0的二级质谱图以及PC、LPC同系物结构通式的质谱碎裂规律

### 2.4 方法学验证

#### 2.4.1 血清样本量

本研究所建立方法面向临床人群队列样本检测，因此在样本量上需充分考量临床样本的特殊性，检测方法对样本量的需求越小，其临床实用价值越突出。样本量小一方面可最大限度减少对患者的创伤与取样负担；更为关键的是样本量的节省是维持临床队列研究延展性的基础保障。

本研究针对上述需求，通过系统优化样本前处理、色谱分离、质谱检测各环节条件，在保证检测性能的前提下，逐步缩减血清样本量。经多次调试验证，当样本量降至10 μL时，仍能维持基线稳定、峰形规整，信号响应能够满足临床检测要求，可以实现小样本量下的高效检测，既契合临床样本珍贵性的保护需求，也为该方法的临床批量应用奠定了基础。对于个别丰度含量较高的同系物，血清样本量还可进一步缩减至1 μL。整个取样过程由自动稀释器全程操作，可保证取样精度和准度。

PC、LPC目标分析物以及内标的色谱图如图4所示，各色谱峰的保留时间与对应物质的关联如表1所示。所有物质在17 min内均洗脱出峰，各峰峰形良好、信噪比佳。本研究所建方法可同时定量分析30种PC、LPC同系物，显著提高了分析效率与临床监测覆盖度。

**图4:**
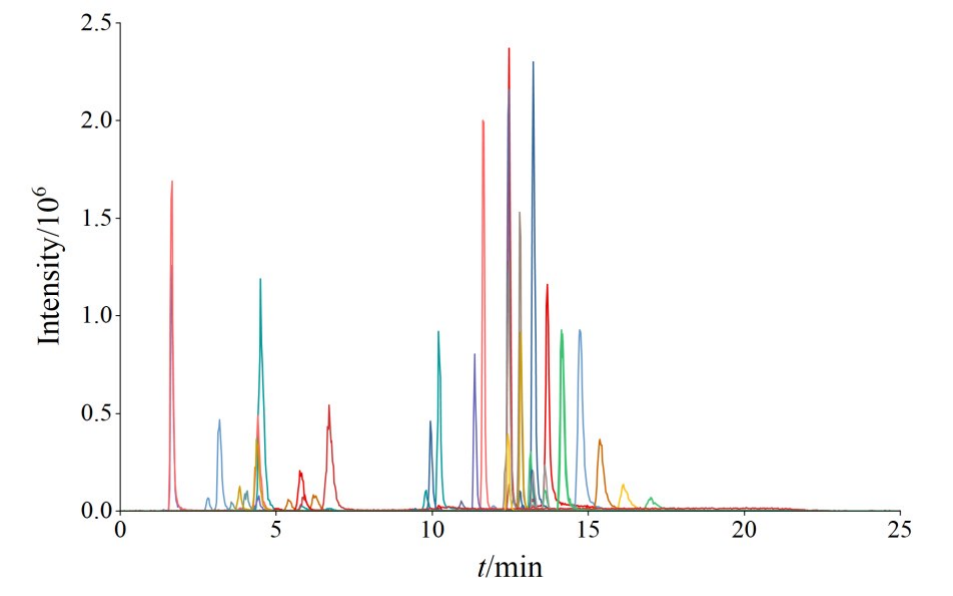
正离子-多反应监测PC、LPC同系物的色谱图

#### 2.4.2 线性关系、检出限和定量限

以PC、LPC质量浓度为*X*，目标物质色谱峰面积与内标峰面积比值为*Y*，进行线性回归分析。稀释混合标准溶液，分别以信噪比为3和10时的质量浓度作为检出限（LOD）和定量限（LOQ）。重复测定5次PC、LPC同系物混合标准溶液，线性范围为0.125~100 μg/mL，平均线性相关系数（*R* ^2^）分别是0.999 7和0.999 9，线性相关性显著。LOD为0.01~1.94 μg/mL，LOQ为0.03~6.48 μg/mL。详细斜率、截距、相关系数、线性范围、LOD和LOQ见表2。

**表2:** PC、LPC同系物的斜率、截距、相关系数、线性范围、检出限和定量限（*n*=5）

Name	Slope	Intercept	*R* ^2^	Linear range/ （μg/mL）	LOD/ （μg/mL）	LOQ/ （μg/mL）
**PC**						
16：0/18：0	0.2791	0.0925	0.9999	0.125-100	0.15	0.56
16：0/16：0	0.2404	0.1827	0.9999	0.125-100	0.04	0.13
16：0/18：2	0.0061	-0.0053	0.9995	0.125-100	1.72	5.74
17：0/17：0	0.2791	0.0925	0.9999	0.125-100	0.15	0.56
18：0/18：0	0.1090	0.1282	0.9999	0.125-100	0.05	0.17
18：1/18：1	0.0645	-0.0564	0.9997	0.125-100	0.13	0.44
18：0/18：1	0.1155	0.0087	0.9999	0.125-100	0.12	0.41
14：1/14：1	0.0804	0.0936	0.9999	0.125-100	0.11	0.42
20：0/20：0	0.0693	0.0724	0.9995	0.125-100	0.05	0.14
9：0/9：0	0.0745	0.0085	0.9999	0.125-100	0.03	0.25
7：0/7：0	0.0279	0.0000	0.9999	0.125-100	0.03	0.28
18：1/14：0	0.1341	0.0380	0.9999	0.125-100	0.02	0.17
18：0/18：2	0.0931	-0.0197	0.9999	0.125-100	0.38	1.26
18：2/18：2	0.0353	-0.0033	0.9998	0.125-100	0.06	0.18
15：0/15：0	0.3274	0.0615	0.9997	0.125-100	0.03	0.09
16：1/16：1	0.1035	-0.0041	0.9997	0.125-100	0.02	0.25
16：0/18：1	0.0417	0.0254	0.9997	0.125-100	0.22	0.73
19：0/19：0	0.0821	0.0821	0.9998	0.125-100	0.08	0.16
21：0/21：0	0.0268	0.1246	0.9992	0.125-100	0.06	0.20
22：0/22：0	0.0124	0.0981	0.9992	0.125-100	0.09	0.17
14：0/14：0	0.0907	0.0964	0.9999	0.125-100	0.04	0.03
14：0/18：0	0.0603	0.1092	0.9998	0.125-100	0.41	1.52
**LPC**						
14：0	0.0800	-0.0007	0.9999	0.125-100	0.08	0.27
15：0	0.0891	0.0191	0.9999	0.125-100	0.09	0.28
16：0	0.2175	0.0404	0.9999	0.125-100	1.94	6.48
18：0	0.2521	0.1359	0.9999	0.125-100	1.03	3.44
20：0	0.1589	0.1430	0.9998	0.125-100	0.07	0.26
24：0	0.2871	0.4933	0.9999	0.125-100	0.01	0.23
18：1	0.2866	-0.1241	0.9999	0.125-100	0.54	1.79

#### 2.4.3 回收率和精密度

向两种血清样本中分别加入5、10、25 μg/mL的混合标准溶液，每种含标样本制备3个平行管，重复测定5个批次。测定加入标准溶液前后的血清样本中PC、LPC同系物的含量，计算加标回收率和相对标准偏差（RSD）以衡量所建方法准确度和精密度，每种物质的回收率和精密度见表3和表4。

**表3:** PC及其同系物在2个样品中的回收率以及精密度

Name	Sample	Added/（μg/mL） （*n*=3）	Found/（μg/mL） （*n*=3）	Recovery/%（*n*=3）	Intra-day RSD/% （*n*=3）	Inter-day RSD/% （*n*=5）	Name	Sample	Added/（μg/mL） （*n*=3）	Found/（μg/mL） （*n*=3）	Recovery/%（*n*=3）	Intra-day RSD/% （*n*=3）	Inter-day RSD/% （*n*=5）
16：0/18：0	S1	5	4.65	91.9	4.0	11.3	18：1/14：0	S1	5	5.07	101.4	2.7	11.3
		10	9.25	92.5	3.3	12.6			10	10.24	102.4	2.6	8.9
		25	22.59	90.4	2.1	9.8			25	25.22	100.9	1.6	10.3
	S2	5	4.90	98.0	2.2	9.1		S2	5	4.90	97.9	2.6	11.2
		10	9.61	96.1	3.7	10.7			10	11.25	112.5	2.5	9.9
		25	21.60	86.5	2.8	10.5			25	25.84	103.3	1.9	10.3
16：0/16：0	S1	5	5.05	100.9	1.3	8.6	18：0/18：2	S1	5	4.73	94.6	2.3	8.1
		10	10.96	109.6	2.4	9.5			10	9.58	95.8	2.4	8.9
		25	24.32	97.3	2.7	8.3			25	24.59	98.4	3.2	10.1
	S2	5	5.06	101.3	3.9	11.8		S2	5	4.91	98.2	2.2	12.1
		10	10.88	108.8	3.1	8.1			10	8.85	88.5	1.2	5.0
		25	25.07	100.3	3.5	9.5			25	25.37	101.5	2.4	9.6
16：0/18：2	S1	5	5.44	108.7	4.6	10.4	18：2/18：2	S1	5	4.81	96.2	3.9	11.3
		10	8.54	85.4	1.3	4.2			10	9.79	97.9	2.6	8.2
		25	25.90	108.0	4.0	11.9			25	22.88	91.5	3.7	10.3
	S2	5	5.39	107.7	2.4	9.8		S2	5	5.05	101.0	1.3	9.9
		10	10.96	109.8	1.3	9.0			10	9.70	97.0	2.4	11.4
		25	27.05	108.2	3.0	14.4			25	23.55	94.2	2.7	9.6
17：0/17：0	S1	5	4.65	91.9	4.0	11.3	15：0/15：0	S1	5	5.10	102.0	3.5	11.0
		10	9.25	92.5	3.3	12.6			10	11.09	110.9	3.3	12.1
		25	22.59	90.4	2.1	9.8			25	23.51	94.0	1.2	6.3
	S2	5	4.90	98.0	2.2	9.1		S2	5	4.60	92.1	3.7	11.7
		10	9.61	96.1	2.7	10.8			10	10.80	108.0	2.2	10.1
		25	21.60	86.5	2.8	10.5			25	22.30	89.2	2.6	9.0
18：0/18：0	S1	5	5.19	103.5	1.7	8.2	16：1/16：1	S1	5	4.29	85.8	2.6	8.4
		10	10.69	106.9	1.2	6.7			10	8.64	86.4	2.1	6.7
		25	25.33	101.3	2.1	7.5			25	23.65	90.5	2.4	8.4
	S2	5	5.25	104.9	3.8	11.5		S2	5	4.92	98.3	1.3	4.6
		10	10.63	106.3	2.4	8.0			10	9.01	90.1	3.2	9.9
		25	25.01	100.0	2.6	7.1			25	21.51	86.1	2.1	8.3
18：1/18：1	S1	5	4.92	98.5	2.3	10.1	16：0/18：1	S1	5	4.88	95.6	2.2	8.1
		10	10.02	100.2	1.3	5.2			10	9.77	97.7	2.9	8.5
		25	24.37	97.5	1.3	7.5			25	26.00	104.0	3.5	9.7
	S2	5	4.97	99.3	2.2	9.0		S2	5	4.99	99.9	2.8	8.8
		10	10.47	104.6	1.1	5.1			10	11.81	114.1	3.3	10.5
		25	23.43	93.7	1.1	5.3			25	24.61	98.4	3.7	9.6
18：0/18：1	S1	5	5.01	100.1	2.3	10.4	19：0/19：0	S1	5	5.14	102.8	2.5	10.3
		10	9.25	92.5	1.1	4.7			10	10.74	107.5	2.1	8.6
		25	22.29	89.1	2.8	10.0			25	25.14	100.5	1.8	5.9
	S2	5	5.05	100.9	4.0	10.4		S2	5	5.19	103.9	3.3	9.6
		10	9.46	94.6	1.7	7.9			10	11.07	110.7	3.0	9.3
		25	21.52	86.1	2.9	9.3			25	24.02	96.1	1.2	4.7
14：1/14：1	S1	5	5.71	114.2	3.3	9.9	21：0/21：0	S1	5	5.05	101.0	2.3	8.4
		10	11.18	111.8	4.6	11.8			10	10.46	104.6	2.2	6.1
		25	27.12	108.5	1.1	3.6			25	26.60	106.4	2.3	7.9
	S2	5	5.42	108.4	2.1	11.3		S2	5	5.54	110.8	3.7	9.5
		10	10.30	102.9	1.4	10.0			10	10.84	108.4	2.1	8.7
		25	25.45	101.8	1.3	8.2			25	26.52	106.1	3.2	9.3
20：0/20：0	S1	5	5.56	111.0	3.3	11.2	22：0/22：0	S1	5	5.33	106.5	3.1	10.6
		10	10.42	104.2	2.2	10.6			10	10.12	101.2	3.3	11.1
		25	24.84	99.4	1.3	10.0			25	26.32	105.3	2.5	10.0
	S2	5	5.04	100.7	1.3	9.9		S2	5	4.93	98.6	4.0	12.4
		10	10.33	103.3	2.6	11.7			10	10.38	103.8	3.3	11.0
		25	25.50	102.0	2.3	10.1			25	26.68	106.7	3.0	9.6
9：0/9：0	S1	5	5.64	112.8	2.1	7.5	14：0/14：0	S1	5	5.00	100.0	3.1	9.5
		10	11.37	113.7	1.5	5.7			10	10.55	105.5	3.6	9.8
		25	26.93	107.7	2.3	7.3			25	25.68	102.7	4.1	11.4
	S2	5	5.58	111.5	2.4	8.5		S2	5	5.19	103.8	2.8	7.5
		10	11.31	113.1	3.7	7.7			10	10.47	104.7	2.1	9.1
		25	27.89	111.6	3.7	9.5			25	26.09	104.3	3.7	9.7
7：0/7：0	S1	5	5.45	109.0	3.0	9.9	14：0/18：0	S1	5	5.36	107.2	2.2	6.0
		10	11.31	113.0	3.6	9.1			10	11.29	112.9	2.3	6.0
		25	26.60	106.4	2.9	9.3			25	28.35	113.4	3.4	11.6
	S2	5	5.52	110.5	2.9	11.6		S2	5	5.64	112.8	2.6	8.8
		10	11.10	111.0	1.6	9.4			10	10.14	113.6	2.3	7.9
		25	25.24	101.0	2.9	9.2			25	28.28	113.1	3.2	7.1

**表4:** LPC及其同系物在2个样品中的回收率和精密度

Name	Sample	Added/（μg/mL） （*n*=3）	Found/（μg/mL） （*n*=3）	Recovery/% （*n*=3）	Intra-day RSD/% （*n*=3）	Inter-day RSD/% （*n*=5）	Name	Sample	Added/（μg/mL） （*n*=3）	Found/（μg/mL） （*n*=3）	Recovery/% （*n*=3）	Intra-day RSD/% （*n*=3）	Inter-day RSD/% （*n*=5）
14：0	S1	5	5.13	102.6	4.4	11.8	20：0	S1	5	5.04	100.8	2.1	9.5
		10	10.35	103.4	3.3	11.5			10	9.98	99.8	1.4	7.5
		25	25.30	101.2	2.1	10.1			25	27.04	108.2	1.5	7.2
	S2	5	5.14	102.8	3.1	10.5		S2	5	5.31	106.1	1.0	7.4
		10	10.37	103.7	1.8	7.2			10	10.98	109.8	2.6	8.9
		25	26.53	106.1	1.1	8.2			25	28.57	114.3	3.8	11.0
15：0	S1	5	5.16	103.2	2.9	8.6	24：0	S1	5	5.22	104.4	2.7	10.4
		10	10.46	104.6	3.3	10.2			10	10.64	106.4	3.2	10.1
		25	25.96	103.8	2.3	7.2			25	25.73	102.9	2.9	7.1
	S2	5	5.14	102.8	3.7	9.7		S2	5	5.34	106.9	2.0	10.4
		10	10.29	102.9	4.1	11.7			10	10.82	108.2	2.6	8.1
		25	26.37	105.5	2.2	11.5			25	27.11	108.4	3.0	9.9
16：0	S1	5	5.43	108.6	3.1	10.2	18：1	S1	5	4.70	94.0	3.7	10.4
		10	10.73	107.2	2.5	7.9			10	10.08	100.4	2.5	7.9
		25	24.86	99.4	2.5	7.0			25	24.71	98.8	2.7	7.6
	S2	5	4.91	98.3	2.7	8.1		S2	5	4.82	96.3	3.1	9.3
		10	9.62	96.2	1.2	6.9			10	9.62	96.2	3.1	11.0
		25	24.89	99.6	2.3	8.3			25	25.70	102.8	2.6	5.6
18：0	S1	5	5.51	110.2	3.5	10.0	18：2	S1	5	4.67	93.4	3.8	9.8
		10	9.94	99.4	2.7	6.6			10	10.06	99.4	2.7	11.1
		25	24.91	99.6	1.1	4.6			25	25.10	99.6	1.5	10.2
	S2	5	4.96	99.2	2.4	10.2		S2	5	4.69	93.8	3.8	7.7
		10	10.77	107.7	4.0	9.3			10	10.10	99.0	2.4	8.6
		25	26.00	104.0	3.5	11.3			25	24.56	102.8	2.5	7.8

由表3和表4可以看出，PC和LPC的回收率为85.4%~114.3%，批内精密度与总精密度的RSD值分别小于4.6%和12.6%，其精密度和回收率均满足临床分析要求^［16］^。这表明，内标物质的使用有助于减少样品前处理过程中的损失干扰，避免对回收率的影响，同时降低误差，从而提高结果的重现性与稳定性。在内标物质选择上，优先采用稳定同位素标记的PC、LPC作为内标；对于无市售同位素内标物的同系物，依据结构相似性及保留时间相近原则，选择相应物质作为内标。

#### 2.4.4 方法的基质效应

我们考察了方法可能存在的离子抑制/增强情况。测定不同保留时间的同位素内标物质在血液基质萃取液和纯溶剂中的响应，其比值百分数定义为基质效应因子。5种内标物质的基质效应因子介于100.0%~108.6%（d4-PC 14：0为108.6%，d9-PC 18：0为100.0%，d5-LPC 15：0为102.4%，d31-LPC 16：0为105.0%，d35-LPC 18：0为108.6%），表明该方法没有明显的离子抑制或增强。此外，我们还采用柱后流动灌注法对潜在基质效应进行了监测。将处理后的血清基质样本通过液相色谱进样，同时利用注射泵将含同位素内标的溶液经三通连接阀注入色谱柱后流出液，使两者混合后进入质谱，监测质谱信号响应变化。结果显示，分析周期内质谱总离子流强度无明显波动，表明所建方法无显著基质效应。

### 2.5 方法的人群应用

为进一步探究本研究建立的分析方法在冠心病风险评估中的应用价值与临床意义。共收集110名接受临床冠状动脉造影术志愿者（男性64名，女性46名，年龄50~70岁），所有研究对象均有明确的临床诊断结论及完整检查资料。采用所建方法定量检测血清中30种PC、LPC同系物。其中29种（详见表1）通过标准曲线定量。LPC 18：2由于没有市售标准品，故采用与其保留时间相近、结构近似且具有标准品的LPC 18：1的标准曲线进行定量。PC在人群中的质量浓度范围为289.96~1 160.88 μg/mL，均值为526.80 μg/mL；LPC在人群中的质量浓度范围为46.07~132.96 μg/mL，均值为73.67 μg/mL。其中，PC 16：0/18：2与 LPC 16：0分别是人血中PC与LPC两类物质中含量占主导地位的优势亚型，二者在人群中的平均质量浓度分别为251.60 μg/mL和50.18 μg/mL。其余PC亚型与LPC亚型的人群浓度分布对比情况如图5所示。

**图5:**
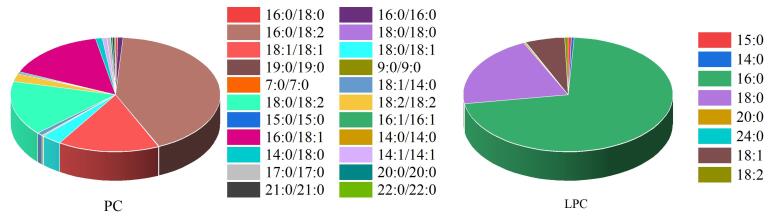
人群中PC、LPC同系物水平分布对比（*n*=110）

鉴于PC、LPC是重要的血清脂质代谢物，且冠状动脉粥样硬化的发生发展与脂质代谢紊乱密切相关，本研究采用所建方法定量检测CAD人群中PC、LPC各同系物水平，通过SPSS 26.0统计学分析软件进行非参数Spearman分析，探究其与CAD临床指标的相关性。与CAD有相关性的PC、LPC同系物结果如图6所示，Total-PC与性别、年龄、Gensini评分及传统冠状动脉疾病血脂危险因素（TC、TG、脂蛋白、胆碱酯酶（ChE））均呈显著正相关（*P*<0.001）；Total-LPC 则与脂代谢指标TC、TG、脂蛋白及ChE呈正相关（*P*<0.01）。上述结果提示，PC、LPC同系物水平的升高对CAD的诊断具有临床参考价值。具体到PC和LPC的不同亚类，PC中的16：0/16：0、16：0/18：2、18：0/18：0、18：1/18：1、18：1/14：0、18：0/18：2、18：2/18：2、15：0/15：0、16：1/16：1、16：0/18：1、14：0/14：0、14：0/18：0以及LPC 18：0、18：1与反映冠状动脉粥样硬化程度的Gensini评分呈显著正相关，而PC 7：0/7：0与之负相关。这一结果提示，不同种类的PC和LPC存在功能异质性。

**图6:**
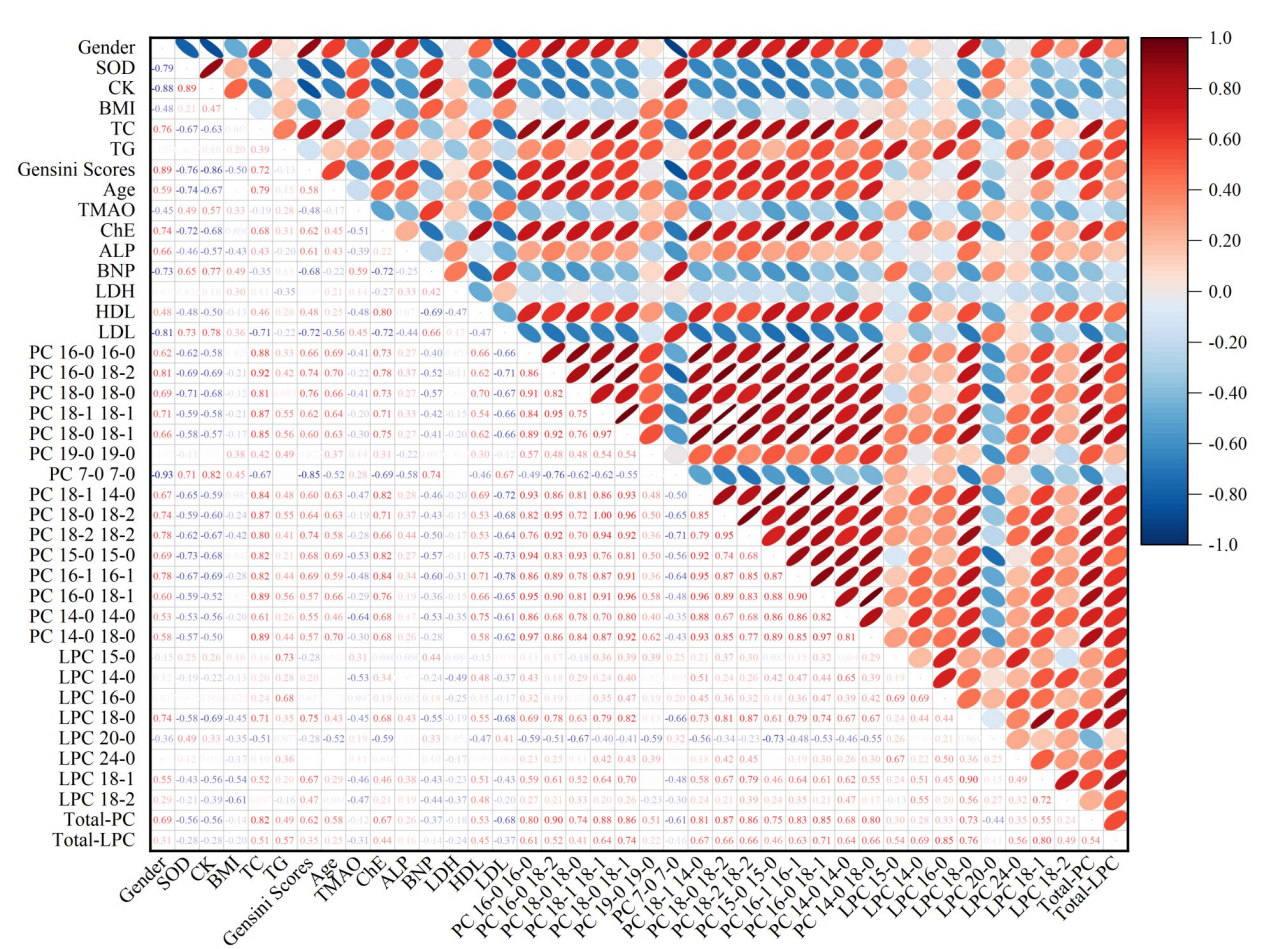
CAD相关PC、LPC同系物水平与临床代谢指标的非参数Spearman相关性热图

本研究依据临床冠状动脉造影诊断结论，将研究对象分为冠心病组（CAD（+））（*n*=55）和对照组（CAD（-））（*n*=55）。对两组人群中与CAD相关的PC、LPC同系物水平进行*t*-检验分析。结果显示，与CAD（-）组相比，CAD（+）组PC 16：0/16：0、16：0/18：2、18：0/18：0、18：1/18：1、18：0/18：1、7：0/7：0、18：1/14：0、18：0/18：2、18：2/18：2、15：0/15：0、16：1/16：1、16：0/18：1、19：0/19：0、14：0/14：0、LPC 15：0、18：0、18：1含量均呈统计学显著性升高，LPC 16：0显著性降低（图7）。不同PC、LPC亚型在CAD中呈现出功能异质性，其机制可能涉及两方面：一方面，PC作为细胞膜的关键组成成分，其代谢异常可破坏血管内皮细胞的结构完整性，延缓脂蛋白的血浆清除过程，使其滞留于血管内皮下，进而加速动脉粥样硬化斑块的形成；作为PC的水解代谢物LPC，则可能通过调节血管平滑肌细胞功能、影响血管张力与弹性，参与CAD的病理进程。同时，PC与LPC的代谢紊乱还可能激活炎症信号通路，促进炎症因子释放，而慢性炎症正是驱动动脉粥样硬化进展的关键因素。另一方面，部分PC、LPC亚类对心血管具有保护作用，其机制可能通过增强卵磷脂胆固醇酰基转移酶活性以参与胆固醇逆向转运、抑制核因子NF-κB等炎症通路，以及调节血管内皮功能来实现^［17，18］^。这提示我们需进一步解析特定亚型在分子层面功能差异的核心驱动机制，同时探索关键亚型作为冠心病风险预测标志物及精准干预靶点的临床转化价值。

**图7:**
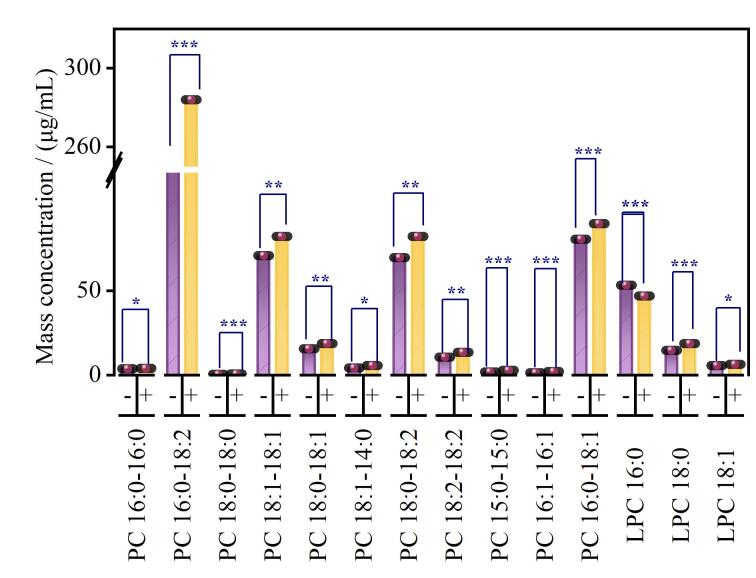
与CAD相关PC、LPC同系物在健康人群和疾病人群中含量比较

## 3 结论

本研究建立了一种基于液相色谱-串联质谱技术测定人血清中 PC、LPC 同系物含量的分析方法。该方法仅需微量血清，一次进样即可实现30种待测物的精确定量。该方法适用于冠心病人群队列研究，为临床精准检测 PC、LPC 同系物提供了简便、精密、覆盖范围广的技术手段。本研究的人群分析结果为血清PC、LPC与冠心病之间的关联提供了新的参考依据，进一步揭示了不同亚类的功能异质性特征，为深入探究不同亚类在冠心病发生发展中的特异性作用机制以及其作为临床标志物的转化应用提供了参考。
